# The economic burden of pain in migraine: a national study of U.S. healthcare expenditures

**DOI:** 10.3389/fneur.2026.1852815

**Published:** 2026-07-22

**Authors:** Monira Alwhaibi, Bander Balkhi, Yazed AlRuthia

**Affiliations:** Department of Clinical Pharmacy, College of Pharmacy, King Saud University, Riyadh, Saudi Arabia

**Keywords:** decomposition analysis, healthcare costs, MEPS, migraine, pain, burden

## Abstract

**Background:**

Migraine is a prevalent and disabling neurological disorder associated with substantial healthcare costs. Coexisting pain is frequently reported among individuals with migraine and may significantly exacerbate both clinical burden and healthcare expenditures. However, limited research has quantified the specific contribution of pain to excess healthcare costs in this population.

**Methods:**

We conducted a cross-sectional analysis using 2019–2022 data from the Medical Expenditure Panel Survey (MEPS) to examine the impact of pain on healthcare expenditures among U.S. adults with migraine. Pain was identified using a self-reported measure of pain interference with daily activities. Individuals were categorized into two groups: migraine with pain and migraine only (without pain). Generalized linear models (GLMs) were used to estimate adjusted healthcare costs, and Blinder–Oaxaca decomposition was applied to assess the extent to which observed cost differences were attributable to patient characteristics.

**Results:**

Among 1,411 adults with migraine, 66% reported pain that interfered with daily function. Individuals with migraine and pain incurred significantly higher total healthcare expenditures than those without pain ($18,748 vs. $10,042; *p* < 0.0001), with outpatient services being the main cost drivers. After adjustment, pain remained significantly associated with higher costs, particularly in outpatient care (incremental cost: $4,546; *p* < 0.0001). Decomposition analysis showed that 76% of the cost difference was explained by observed characteristics, primarily poorer perceived health, unemployment, and comorbid arthritis.

**Conclusion:**

Pain substantially increases the economic burden of migraine, largely through elevated outpatient care costs. These findings highlight the need for comprehensive pain assessment and integrated care strategies to manage pain in individuals with migraine and reduce healthcare expenditures.

## 1 Introduction

Migraine is a highly prevalent, debilitating neurological disorder affecting approximately 11.7%–14.7% of the U.S. population, with prevalence rates nearly twice as high in women compared to men (1). Although overall prevalence has remained steady, the proportion of individuals experiencing moderate-to-severe migraine-related disability has risen sharply from 22.0% in 2005 to over 42% by 2018 (1). Globally, the burden of migraine has increased substantially from 1990 to 2021, with prevalence rising by 58% and disability showing a similar upward trend (2). While women continue to bear a greater overall burden, the rate of increase has been faster among men, and projections suggest the global prevalence will continue to rise through 2050 (2). Globally, migraine remains the second leading cause of disability, as reported by the Global Burden of Disease Study 2019 (3). Patients with migraine incur significantly higher direct and indirect annual healthcare costs and health services utilization compared to those without migraine, with prescription drugs accounting for nearly half of the total expenses (4–6). Migraine is also associated with poorer health-related quality of life, and productivity losses (7, 8).

In addition to the burden of migraine itself, pain is common among adults with migraine and may significantly intensify the clinical, economic, and social burden of this disorder (9, 10). Evidence suggests that individuals with migraine are more likely to report coexisting pain compared to those without migraine. A review of published studies reported that over 40% of individuals with migraine reported having at least one other chronic pain condition (11). These pain comorbidities are not merely co-occurring they are synergistic, contributing to increased headache frequency, greater pain severity, and worse health outcomes (11).

Importantly, coexisting pain is associated with higher healthcare utilization and greater medical costs among people with migraine (12). An analysis of database demonstrated that individuals with both migraine and chronic pain had significantly higher healthcare costs compared to those with migraine alone (12). Moreover, pain comorbidities are associated with more frequent use of pain killer medications such as opioids, non-steroidal anti-inflammatory drugs (NSAIDs), and complementary therapies, further increasing the costs of migraine.

Despite the high economic burden of pain among adults with migraine, few studies have systematically examined how coexisting pain contributes to excess healthcare costs within this population. Most cost-of-illness studies have focused on the direct burden of migraine, while underestimating or ignoring the additive costs of chronic pain conditions. Understanding the specific contribution of pain to healthcare expenditures is crucial for improving care strategies and guiding resource allocation. This study aims to fill this critical gap by examining the impact of pain on healthcare costs among adults with migraine. We’ll use nationally representative data from the Medical Expenditure Panel Survey (MEPS) to achieve this. Specifically, we compare healthcare expenditures between individuals with migraine who report pain and those who do not, after adjusting for demographic, socioeconomic, and health status characteristics. By quantifying the incremental cost burden of pain among people with migraine, this study provides important insights for clinicians, policymakers, and health systems working to reduce costs and improve outcomes in adults with migraine.

## 2 Methods

### 2.1 Study population and data

A cross-sectional study was conducted using retrospective data from MEPS, a nationally representative survey of the United States (US) funded by the Agency for Healthcare Research and Quality. Eligible adults are aged 18 years and above, those with Migraine, who are alive within the study years, and have no missing data on pain. We utilized data from the 2019–2022 Medical Expenditure Panel Survey (MEPS), including files on households, medical conditions, prescribed medications, outpatient visits, and other related files. MEPS surveys approximately 30,000 individuals annually from a nationally representative U.S. sample. For this analysis, after restricting to adults with migraine and excluding records with missing pain data, the final sample was 1,411. Migraine cases in MEPS are identified based on self-reported medical conditions that are subsequently coded by professional coders using ICD-10-CM codes. The survey does not differentiate between episodic and chronic migraine.

### 2.2 Measures

#### 2.2.1 Outcome: healthcare expenditures

The analysis encompassed multiple types of healthcare expenditures, including inpatient care, outpatient services, prescription medications, emergency department visits, and other healthcare-related costs (e.g., dental services, vision care, and durable medical equipment). Aggregate healthcare expenditures were computed as the sum of all expenditure categories. To account for inflation, all expenditure values were adjusted using the Consumer Price Index and standardized to 2022 constant U.S. dollars, as reported by the U.S. Bureau of Labor Statistics (13).

#### 2.2.2 Key independent variable (pain)

Data on pain were obtained from the MEPS. Pain was identified in MEPS using the variable ADPAIN42 (14), which captures whether respondents reported that pain interfered with their normal work outside the home and housework during the past 4 weeks. The ADPAIN42 variable captures interference from pain in daily activities during the past 4 weeks, regardless of source. Thus, it reflects overall pain burden and not specifically migraine-related pain. The original responses, which included the options “*not at all*,” “*a little bit*,” “*moderately*,” “*quite a bit*,” and “*extremely*,” were recoded into two categories: (1) Pain (Combining *a little bit, moderately*, *quite a bit* and *extremely*), and (2) No pain (*not at all*). Based on the presence of documented pain, adults with migraine were categorized into two groups: (1) Migraine with pain and (2) Migraine only (i.e., without pain). This approach of identifying pain has been used by previous studies using MEPS data (15–19). Compared with diagnosis-based approaches that rely on specific ICD-10 codes, this measure identifies individuals experiencing clinically meaningful pain that interferes with daily activities, including cases that may not be coded as chronic pain conditions.

#### 2.2.3 Other independent variables

Additional covariates included demographic, socioeconomic, and health-related factors such as sex, race/ethnicity, age, marital status, geographic region, employment status, educational attainment, income level, self-reported physical health, health insurance status, prescription drug coverage, and physical activity.

Furthermore, we assessed the presence of a range of common chronic conditions known to influence healthcare utilization and costs. These included: cardiovascular disease, hypertension, diabetes, hyperlipidemia, asthma, chronic obstructive pulmonary disease (COPD), gastroesophageal reflux disease (GERD), arthritis, thyroid disorders, cancer, anxiety, and depression.

### 2.3 Statistical analyses

Descriptive statistics were used to characterize the study population across two Migraine groups (Migraine with pain, and Migraine only). Differences in unadjusted mean total and type of healthcare expenditures among migraine groups were assessed using one-way analysis of variance (ANOVA). To evaluate the association between pain and healthcare expenditures among individuals with migraine, generalized linear models (GLMs) with a log link and gamma distribution were employed. These models adjusted for a comprehensive set of covariates, including sociodemographic variables, insurance status, health behaviors, self-perceived physical health, and other chronic health conditions. GLMs were chosen over ordinary least squares (OLS) regressions of log-transformed costs due to their ability to address heteroscedasticity and eliminate retransformation bias. To further quantify the contribution of pain to differences in healthcare expenditures, a linear decomposition technique based on the Blinder–Oaxaca framework was applied. This approach decomposes the observed differences in average expenditures between groups (e.g., those with and without pain) into components attributable to differences in observable characteristics (explained portion) and differences in coefficients or returns to those characteristics (unexplained portion). Statistical significance was defined as a *p*-value < 0.05. All analyses accounted for the complex survey design of MEPS by incorporating strata, primary sampling units, and person-level weights. Analyses were conducted using the survey procedures in SAS 9.4 (SAS Institute Inc., Cary, NC, USA) and STATA 15.1 (StataCorp LLC, College Station, TX, USA).

## 3 Results

### 3.1 Characteristics of the study sample

Table 1 presents the demographic, socioeconomic, and health-related characteristics of our study sample. Among 1,411 adults with migraine, the majority (66.0%) reported pain. Pain was significantly more common among older adults, especially those aged 50–64 (74.7%) and >65 (73.1%) compared to those aged 18–39 (57.0%; *p* < 0.0001). Pain prevalence was higher among poor individuals (80.1%, *p* < 0.0001), the unemployed (79.2%, *p* < 0.0001), those with public insurance (80.6%, *p* < 0.0001), and individuals without prescription drug coverage (77.1%, *p* < 0.0001). Additionally, those with fair/poor self-reported health had notably higher pain prevalence (92.5%) compared to those in excellent/very good health (48.3%, *p* < 0.0001). Physical inactivity was also associated with pain (71.0% vs. 59.1%, *p* < 0.0001).

**Table 1:** Characteristics of the study sample, number and weighted row percentage of characteristics by pain among adults with migraine.

	Total sample		Pain		No pain		*χ* ^2^	*p*-value	Sig.
	*N*	Wt.%	*N*	Wt.%	*N*	Wt.%			
All	1,411	100.0	954	66.0	457	34.0			
Age in years									
18–39	337	28.7	188	57.0	149	43.0	22.412	<0.0001	***
40–49	288	21.9	173	59.2	115	40.8			
50–64	484	32.5	369	74.7	115	25.3			
>65	302	16.9	224	73.1	78	26.9			
Gender									
Women	1,198	85.0	818	66.6	380	33.4	0.723	0.395	
Men	213	15.0	136	62.3	77	37.7			
Race/ethnicity									
White	1,023	77.1	688	66.0	335	34.0	6.253	0.100	
African American	138	7.6	104	73.3	34	26.7			
Latino	161	8.1	94	54.3	67	45.7			
others	89	7.2	68	71.0	21	29.0			
Marital status									
Married	723	56.8	439	61.3	284	38.7	22.933	<0.0001	***
Widow	428	24.9	350	80.4	78	19.6			
Sep/Div	260	18.3	165	60.7	95	39.3			
Education level									
<High school	44	1.9	35	78.9	9	21.1	7.370	0.061	
High school	82	4.4	69	80.2	13	19.8			
>High school	1,280	93.6	847	65.1	433	34.9			
Region									
Northeast	242	15.7	154	61.7	88	38.3	1.417	0.702	
Mid-west	348	24.7	238	66.6	110	33.4			
South	476	37.1	336	67.9	140	32.1			
West	345	22.6	226	64.9	119	35.1			
Employment									
Employed	779	62.5	448	58.0	331	42.0	37.328	<0.0001	***
Not employed	632	37.5	506	79.2	126	20.8			
Poverty status									
Poor	225	11.9	183	80.1	42	19.9	31.875	<0.0001	***
Near poor	237	14.4	184	76.4	53	23.6			
Middle income	375	26.6	264	71.4	111	28.6			
High income	574	47.1	323	56.1	251	43.9			
Health insurance									
Private	900	71.5	543	60.3	357	39.7	36.791	<0.0001	***
Public	489	27.0	397	80.6	92	19.4			
Uninsured	22	1.4	14	74.9	8	25.1			
Rx insurance									
Rx insurance	748	61.2	438	58.8	310	41.2	33.621	<0.0001	***
No Rx insurance	663	38.8	516	77.1	147	22.9			
General health									
Ex/very good	567	43.5	271	48.3	296	51.7	149.235	<0.0001	***
Good	430	30.5	304	68.5	126	31.5			
Fair/poor	414	26.0	379	92.5	35	7.5			
Physical activity									
Three times/week	585	42.5	352	59.1	233	40.9	17.049	<0.0001	***
No exercise	821	57.3	599	71.0	222	29.0			
Heart									
Yes	138	8.3	117	82.8	21	17.2	9.106	0.003	**
No	1,273	91.7	837	64.4	436	35.6			
Hypertension									
Yes	468	27.9	376	78.4	92	21.6	23.614	<0.0001	***
No	943	72.1	578	61.1	365	38.9			
Diabetes									
Yes	192	10.5	157	79.4	35	20.6	7.699	0.006	**
No	1,219	89.5	797	64.4	422	35.6			
Hyperlipidemia									
Yes	400	25.2	310	77.7	90	22.3	17.846	<0.0001	***
No	1,011	74.8	644	62.0	367	38.0			
Asthma									
Yes	266	17.2	216	77.6	50	22.4	8.805	0.003	**
No	1,145	82.8	738	63.5	407	36.5			
COPD									
Yes	118	7.9	95	76.9	23	23.1	2.738	0.098	
No	1,293	92.1	859	65.0	434	35.0			
Arthritis									
Yes	250	15.3	223	89.2	27	10.8	64.906	<0.0001	***
No	1,161	84.7	731	61.7	430	38.3			
GERD									
Yes	297	19.6	234	78.2	63	21.8	17.490	<0.0001	***
No	1,114	80.4	720	63.0	394	37.0			
Cancer									
Yes	133	8.9	101	73.1	32	26.9	2.569	0.109	
No	1,278	91.1	853	65.3	425	34.7			

Based on 1,411 individuals with migraine, aged 18 years and older and were alive during the calendar years 2019, 2020, 2021, and 2022.

Wt = weighted; Rx = Medication; Wid./Div./Sep. = widowed, divorced, and separated; COPD = chronic obstructive pulmonary disease; GERD = gastroesophageal reflux disease.

Pain was significantly more prevalent among individuals with chronic conditions. High rates were observed among those with arthritis (89.2%, *p* < 0.0001), hypertension (78.4%, *p* < 0.0001), diabetes (79.4%, *p* = 0.006), hyperlipidemia (77.7%, *p* < 0.0001), GERD (78.2%, *p* < 0.0001), asthma (77.6%, *p* = 0.003), and heart disease (82.8%, *p* = 0.003).

Conversely, no statistically significant differences in pain status were found by gender (*p* = 0.395), race/ethnicity (*p* = 0.100), region (*p* = 0.702), education level (*p* = 0.061), COPD (*p* = 0.098), or cancer (*p* = 0.109).

### 3.2 Mean total and type of healthcare expenditures by migraine group

Table 2 illustrates the mean healthcare expenditures for adults with migraine, stratified by pain status. Adults with migraine and functionally significant pain had significantly higher total healthcare expenditures compared to those without pain ($18,748 vs. $10,042, *p* < 0.0001). This cost difference was primarily driven by significantly greater spending in outpatient care ($7,670 vs. $3,605, *p* < 0.0001), emergency room services ($6,651 vs. $3,130, *p* < 0.0001), prescription medications ($650 vs. $272, *p* = 0.0002), and other healthcare services such as dental, vision, and durable medical equipment ($1,480 vs. $960, *p* < 0.0001).

**Table 2:** Mean healthcare expenditures by pain among individuals with migraine.

	Migraine and pain (*N* = 954)		Migraine only (*N* = 457)		*p*-value
	Mean ($)	SE	Mean ($)	SE	
Expenditures					
Total	18,748.17	1,140.27	10,042.14	1,046.17	<0.0001
Inpatient	2,297.00	307.63	2,075.80	740.10	0.1097
Outpatient	7,670.23	523.73	3,604.61	319.67	<0.0001
Prescription	650.07	65.70	271.67	50.88	0.0002
Emergency room	6,651.19	833.58	3,130.47	529.62	<0.0001
Other	1,479.68	113.74	959.60	167.68	<0.0001

Unadjusted weighted mean expenditures based on 1,411 individuals with Migraine, aged 18 years and older and were alive during the calendar years. Medical Expenditure Panel Survey data of 2019, 2020, 2021, and 2022.

Other expenditures included dental, vision, durable medical equipment use, and others.

SE = standard error.

While mean inpatient expenditures were slightly higher among those with pain ($2,297 vs. $2,076), this difference was not statistically significant (*p* = 0.1097). These findings suggest that pain among individuals with migraine is associated with a substantial increase in overall and type of healthcare costs, particularly in outpatient and emergency services.

### 3.3 Adjusted total and type of healthcare expenditures by migraine group

Table 3 presents the adjusted healthcare expenditures. After adjusting for sociodemographic, health, and insurance covariates using a Generalized Linear Model with log link and gamma distribution, individuals with migraine and pain had significantly higher total healthcare expenditures compared to those with migraine only. The incremental cost associated with functionally significant pain was $4,110 (*β* = 0.218, *p* = 0.024; 95% CI: 0.029–0.407).

**Table 3:** Intercept and parameter estimates for the pain groups from adjusted generalized linear model with log link and gamma distribution on healthcare expenditures.

Migraine and pain								
Expenditures	Intercept	SE	*β*	SE	Incremental cost	Lower CI	Upper CI	*p*-value
Total	10.477	0.425	0.218	0.096	$4,109.9	0.029	0.407	0.024
Inpatient	5.863	1.593	0.018	0.343	$42.8	−0.659	0.696	0.957
Outpatient	9.300	0.404	0.509	0.094	$4,545.7	0.324	0.694	<0.0001
Prescription	10.520	0.776	0.029	0.170	$170.8	−0.306	0.365	0.863
Emergency	9.785	1.147	0.337	0.232	$220.9	−0.122	0.795	0.149
Other	8.318	0.610	0.010	0.146	$13.6	−0.279	0.298	0.947

Based on 1,411 adults with Migraine, aged 18 years and older and were alive during the calendar years of 2019, 2020, 2021, and 2022.

Other expenditures included dental, vision, durable medical equipment use, and others.

Reference group for migraine groups = migraine only.

*β* = coefficient; SE = standard error.

The significant cost difference was in outpatient expenditures, with an incremental cost of $4,546 (*β* = 0.509, *p* < 0.0001). In contrast, other expenditure categories such as inpatient, prescription drugs, emergency care, and other services showed no statistically significant differences. This indicates that the main driver of higher overall costs in the pain group is outpatient care.

### 3.4 Factors explaining excess pain related healthcare expenditure among adults with migraine from linear decomposition analysis

The Blinder–Oaxaca decomposition analysis (Table 4) revealed that adults with migraine and pain incurred significantly higher total healthcare expenditures than those with migraine only, with a mean difference of $9,392 (*p* < 0.0001). Of this difference, $7,135 (76%) was explained by observable characteristics, while $2,904 (31%) remained unexplained and was not statistically significant (*p* = 0.059).

**Table 4:** Blinder–Oaxaca decomposition of log-transformed total healthcare differences by pain.

	*β*	SE	*p*-value	Lower CI	Upper CI
Migraine and pain	19,885.5	954.6	<0.0001	18,014.5	21,756.5
Migraine only	10,493.2	883.3	<0.0001	8,762.0	12,224.4
Difference	9,392.3	1,300.6	<0.0001	6,843.2	11,941.4
Explained	7,134.6	1,274.3	<0.0001	4,636.9	9,632.3
Unexplained	2,903.6	1,539.3	0.059	−113.4	5,920.5
Interaction	−645.9	1,564.4	0.68	−3,712.1	2,420.3
Estimate due to difference in characteristics (explained)					
Age group	260.0	386.7	0.501	−497.9	1,017.8
Gender	24.2	63.6	0.703	−100.5	148.9
Race	−0.2	14.3	0.991	−28.3	28.0
Marital status	281.1	173.7	0.106	−59.4	621.7
Education	−297.8	230.3	0.196	−749.3	153.7
Employment	1,339.0	586.4	0.022	189.7	2,488.3
Income	−655.5	521.1	0.208	−1,676.8	365.9
Health insurance	−39.6	564.4	0.944	−1,145.7	1,066.5
RX insurance	−37.2	608.7	0.951	−1,230.3	1,155.9
Perceived health	3,484.2	1,005.7	0.001	1,513.1	5,455.3
Physical activity	261.6	244.5	0.285	−217.6	740.7
Region of residence	−48.2	99.0	0.626	−242.2	145.8
Heart disease	178.7	319.4	0.576	−447.2	804.7
Hypertension	567.3	457.2	0.215	−328.7	1,463.3
Diabetes	−123.8	290.2	0.67	−692.6	444.9
Hyperlipidemia	−282.8	325.0	0.384	−919.8	354.2
Asthma	175.4	331.8	0.597	−474.9	825.8
COPD	350.6	218.1	0.108	−76.9	778.0
Arthritis	1,723.9	684.5	0.012	382.3	3,065.5
GERD	−26.5	280.2	0.925	−575.7	522.8

Results based on Blinder–Oaxaca decomposition.

Among the explanatory variables, the most substantial contributors were perceived health status (*$3,484; p* = 0.001), employment status (*$1,339; p* = 0.022), and arthritis comorbidity (*$1,724; p* = 0.012). Other variables including age, marital status, and chronic conditions (e.g., hypertension, COPD) had smaller, non-significant contributions. This suggests that worse perceived health, unemployment, and arthritis play key roles in driving the cost differences associated with pain among adults with migraine.

## 4 Discussion

This study highlights the substantial economic burden associated with pain among adults with migraine in the United States. Using nationally representative MEPS data, we found that approximately two-thirds of adults with migraine reported pain that interfered with their daily activities. This high prevalence emphasizes the frequent coexistence of pain among individuals with migraine, previous findings showing that migraine patients experience additional chronic pain conditions such as arthritis, neck pain, or fibromyalgia (20). This reflects the broader clinical landscape in which migraine rarely exists in isolation. Comorbid chronic pain conditions are common and have been associated with significantly worse outcomes, including more severe pain-related physical limitations, impaired psychosocial functioning, increased health-care utilization, and a greater likelihood of adverse life experiences (21). In light of these broader clinical impacts, it is important to consider how pain was evaluated in this study. We used a single self-reported item that reflects the extent to which pain interferes with normal daily activities, which likely captures pain from multiple sources, not only migraine. This broader definition provides valuable insight into the cumulative burden of pain among individuals with migraine. While this may not fully capture the complexity of pain or its clinical source, it serves as a valuable proxy for identifying individuals experiencing functionally significant pain. This approach reflects a real-world perspective and aligns with prior research showing that coexisting pain conditions frequently accompany migraine and are among the most measured health outcomes in migraine clinical trials (22). Further, because the pain measure assessed interference during the previous 4 weeks, some respondents may have reported pain related to a recent migraine episode rather than a distinct chronic pain condition. Consequently, the observed prevalence of pain may reflect both coexisting pain disorders and residual pain-related disability associated with migraine attacks. The findings should therefore be interpreted as representing the burden of functionally significant pain among individuals with migraine. In addition, our findings revealed that pain was significantly more prevalent among older adults, the unemployed, those living in poverty, and individuals with poor self-reported health and chronic conditions. These findings highlight the intersection of pain with socioeconomic disadvantage and comorbidities.

Building on this understanding of pain prevalence, our analysis further revealed a significant association between the presence of pain and higher healthcare expenditures compared to those without pain. Specifically, the average total healthcare spending was higher for those with functionally significant pain, with outpatient visits accounting for the largest share of this difference. After adjusting for relevant sociodemographic and health factors, the presence of pain remained significantly associated with increased costs, particularly in outpatient care. Several factors may explain this finding. First, individuals experiencing pain whether migraine-related or from comorbid conditions are more likely to engage with outpatient services for ongoing management, including diagnostic evaluations, pain consultations, and physical therapy (17). Unlike inpatient or emergency care, outpatient settings are more accessible and better suited for the longitudinal management of chronic pain. This pattern aligns with prior studies that have shown outpatient visits as the dominant cost driver in chronic pain populations (23). These findings underscore the additive economic impact of pain on top of the already high healthcare burden of migraine.

To better understand the drivers of this increased expenditure, we conducted a decomposition analysis, which revealed that approximately two-thirds of the excess healthcare costs associated with pain were attributable to observable characteristics, notably poorer perceived health, unemployment, and comorbid arthritis. These factors likely reflect more complex clinical profiles and greater care needs among adults with both migraine and pain. However, a portion of the cost difference remained unexplained, suggesting that unexplained component of the expenditure difference likely reflects factors not captured in MEPS, including migraine severity, duration and chronicity of pain, treatment complexity, provider prescribing and referral patterns, access to specialty care, patient healthcare-seeking behavior, and variation in clinical management approaches. These factors may contribute substantially to healthcare expenditures beyond the demographic and clinical characteristics included in our model.

### 4.1 Implications

#### 4.1.1 Clinical implications

Given that outpatient care is the primary driver of excess costs, clinicians should prioritize comprehensive pain assessment. The findings of this study also underscore the need for healthcare providers to screen for pain, especially in older adults, those with poor physical health, and individuals with multiple chronic conditions can aid in early identification and more effective care planning.

#### 4.1.2 Public health implications

At the population level, the high prevalence of self-reported pain among individuals with migraine highlights a significant public health concern. Pain not only exacerbates disability but also amplifies healthcare system strain through increased utilization. Public health campaigns should focus on raising awareness of migraine-pain comorbidities, improving pain literacy among the migraine patients and healthcare professionals, and promoting preventive strategies for chronic pain in susceptible populations.

#### 4.1.3 Policy implications

The results support the need for policy initiatives that expand access to affordable outpatient and pain management services, particularly for socioeconomically vulnerable groups. Policymakers should consider integrating pain and migraine management guidelines into existing chronic disease management, and provide incentives to healthcare models that optimize pain management and outpatient service delivery that drive systemic improvements and cost efficiencies. This could include bundled payments for integrated pain programs or enhanced reimbursement for multidisciplinary pain clinics. Because arthritis emerged as one of the strongest contributors to excess healthcare expenditures, integrated care models should extend beyond general pain management and specifically promote collaboration between neurology and rheumatology services. Multidisciplinary clinics that coordinate migraine management with evaluation and treatment of musculoskeletal disorders may help reduce fragmented care, improve symptom control, and decrease outpatient healthcare utilization. Policymakers should consider reimbursement models that support coordinated neurologic-rheumatologic care pathways for patients with migraine and coexisting pain conditions

#### 4.1.4 Research implications

Future research should explore the causal pathways linking migraine, pain, and healthcare expenditures using longitudinal data. There is also a need to investigate intervention strategies that reduce pain-related disability and costs, especially among subgroups identified as high-risk in this study (e.g., older adults, the unemployed, and those with multiple chronic conditions). Further studies should also evaluate the long-term impact of integrated care models for individuals with migraine and functionally significant pain, including their effects on patient outcomes and overall healthcare spending. Qualitative research could also explore patient and provider experiences with coexisting pain to inform intervention development.

### 4.2 Study limitations

Several limitations should be acknowledged. First, pain was self-reported and not specifically linked to migraine attacks, which may lead to misclassification. Nonetheless, prior research suggests that pain-related disability is common in individuals with migraine, regardless of whether it is attributed to migraine itself or to comorbid conditions. Second, the cross-sectional design limits its ability to draw causal inference. Bedsides, a key limitation is that the MEPS variable used does not distinguish migraine-specific pain from pain due to other conditions. Therefore, our findings represent the cumulative burden of coexisting pain rather than migraine-related pain alone. Another major limitation is the inability to distinguish episodic from chronic migraine. Chronic migraine is associated with greater disability, more frequent healthcare utilization, and higher costs, and is also more likely to coexist with pain. Besides, some of the excess expenditures attributed to pain may reflect unmeasured migraine severity; however, MEPS does not provide detailed information on headache frequency, attack duration, or migraine severity, or subtypes, therefore, we were unable to perform sensitivity analyses to evaluate this potential confounding. Finally, although outpatient expenditures were the main driver of excess costs, we were unable to identify the specific outpatient services or provider specialties responsible for these expenditures. Future studies using event-level data or claims databases may help clarify the outpatient cost drivers among adults with migraine and pain. Despite these limitations, the findings are robust due to the nationally representative sample, the use of inflation-adjusted cost data, and the application of advanced modeling and decomposition techniques.

## 5 Conclusion

In conclusion, pain significantly amplifies the economic burden of migraine among U.S. adults, largely through increased outpatient care costs. These results emphasize the need for comprehensive pain assessment and management strategies in individuals with migraine, and call for integrated care models that address coexisting pain to reduce healthcare costs and improve patient outcomes.

## Data Availability

The datasets presented in this study can be found in online repositories. The names of the repository/repositories and accession number(s) can be found at: https://meps.ahrq.gov.
